# Diabetic vascular diseases: molecular mechanisms and therapeutic strategies

**DOI:** 10.1038/s41392-023-01400-z

**Published:** 2023-04-10

**Authors:** Yiwen Li, Yanfei Liu, Shiwei Liu, Mengqi Gao, Wenting Wang, Keji Chen, Luqi Huang, Yue Liu

**Affiliations:** 1 National Clinical Research Center for Chinese Medicine Cardiology, Xiyuan Hospital, Chinese Academy of Chinese Medical Sciences, Beijing, 100091 China; 2 The Second Department of Gerontology, Xiyuan Hospital, China Academy of Chinese Medical Sciences, Beijing, 100091 China; 3 Department of Nephrology and Endocrinology, Wangjing Hospital, China Academy of Chinese Medical Sciences, Beijing, 100102 China; 4 China Center for Evidence-based Medicine of TCM, China Academy of Chinese Medical Sciences, Beijing, 100010 China

**Keywords:** Endocrine system and metabolic diseases, Metabolic disorders, Cardiology, Molecular medicine

## Abstract

Vascular complications of diabetes pose a severe threat to human health. Prevention and treatment protocols based on a single vascular complication are no longer suitable for the long-term management of patients with diabetes. Diabetic panvascular disease (DPD) is a clinical syndrome in which vessels of various sizes, including macrovessels and microvessels in the cardiac, cerebral, renal, ophthalmic, and peripheral systems of patients with diabetes, develop atherosclerosis as a common pathology. Pathological manifestations of DPDs usually manifest macrovascular atherosclerosis, as well as microvascular endothelial function impairment, basement membrane thickening, and microthrombosis. Cardiac, cerebral, and peripheral microangiopathy coexist with microangiopathy, while renal and retinal are predominantly microangiopathic. The following associations exist between DPDs: numerous similar molecular mechanisms, and risk-predictive relationships between diseases. Aggressive glycemic control combined with early comprehensive vascular intervention is the key to prevention and treatment. In addition to the widely recommended metformin, glucagon-like peptide-1 agonist, and sodium-glucose cotransporter-2 inhibitors, for the latest molecular mechanisms, aldose reductase inhibitors, peroxisome proliferator-activated receptor-γ agonizts, glucokinases agonizts, mitochondrial energy modulators, etc. are under active development. DPDs are proposed for patients to obtain more systematic clinical care requires a comprehensive diabetes care center focusing on panvascular diseases. This would leverage the advantages of a cross-disciplinary approach to achieve better integration of the pathogenesis and therapeutic evidence. Such a strategy would confer more clinical benefits to patients and promote the comprehensive development of DPD as a discipline.

## Introduction

Diabetes mellitus (DM) and its complications pose a serious threat to human health and have become a global public health issue.^1,2^ Over 90% of patients with diabetes have type 2 DM (T2DM).^3,4^ Diabetic complications can be classified according to the involvement of cardiopathy and encephalopathy, nephropathy, retinopathy, and peripheral vasculopathy.^5–7^ DM increases the risk of all these complications, and multiple vasculopathy is associated with a poorer prognosis.^8^ Recent intensive investigations into diabetic complications have significantly promoted the understanding of the pathogenesis of this disease. However, the increasing division of medical science into various subspecialties, has resulted in a tendency to focus on localized lesions instead of integrating overall evidence. Thus, a holistic investigation of diabetic complications involving multiple systems and different angiopathies is needed.

The pathology of diabetic complications has a high degree of commonality at the vascular level; that is, complications manifest mostly as endothelial dysfunction and atherosclerosis (AS).^9^ DM being a risk factor for vascular disease, the several vascular comorbidities seriously affect the prognosis and treatment of patients, leading to the concept of “panvascular disease”.^10–12^ Since the late 20th century, the concept of a “vessel tree”^13^ has been proposed and “polyvascular atherosclerotic disease”^14^ has been defined considering coronary and non-coronary AS, mainly peripheral arterial and cerebrovascular diseases (peripheral vascular disease and cerebrovascular disease respectively). This definition indicates that comprehensive management of the multivessel disease is clinically essential for improving outcomes and prognoses. However, this definition does not consider either microvascular disease (especially in vital organs) or multidisciplinary fusion. To improve this definition, we propose the concept of diabetic panvascular disease (DPD). This is a clinical syndrome in which AS is a common pathology between macrovessels and microvessels in the cardiac, cerebral, renal, ophthalmic, and peripheral systems in patients with diabetes. The main outcomes would be cardiovascular and cerebrovascular events, and the prognosis could be improved through aggressive intervention against metabolic abnormalities.

Diabetic complications are usually classified in two dimensions: macro/microvascular disease, or complications classified by target organs. DPDs synthesize these concepts. This article systematically reviews general pathological manifestations of vascular lesions and differences in the etiology of macro/microvascular lesions; pathological manifestations and molecular mechanisms of different target organs in DPDs; common molecular mechanisms and therapeutic targets in DPDs; time course characteristics of pathological changes in organs and mutual predictive effects among DPDs to provide clues for early diagnosis. Our findings should promote the establishment of a multidisciplinary DPD management system.

### Diabetes and panvasculopathy

**Fig. 1:**
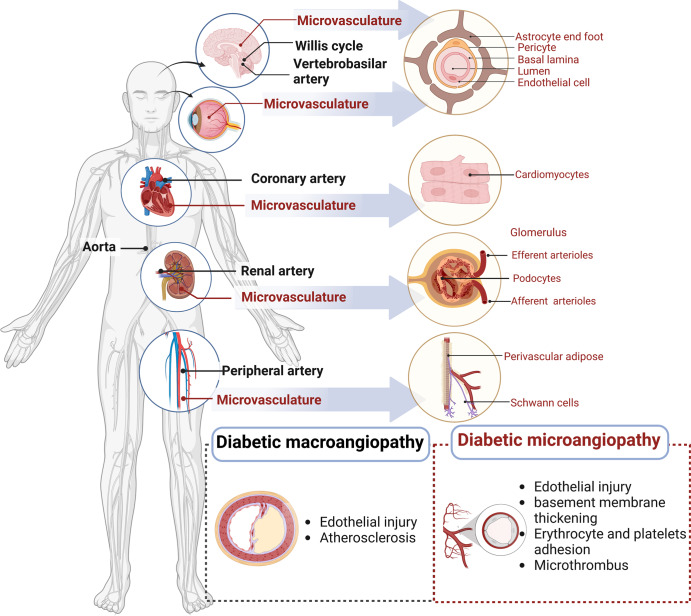
Schematic overview of panvasculopathy in diabetes mellitus. Diabetic panvasculopathy involves the cardiac, cerebral, renal, ophthalmic and peripheral systems. The macrovascular lesions are in black text. The microvascular lesions are in red. The microvascular system varies in different organs, which affects vascular function

The vasculature comprises endothelial cells (EC), smooth muscle cells (SMC), pericytes, fibroblasts, and various other types of cells. AS, endothelial barrier damage, loss of pericytes, capillary thinning, and angiogenic disorders are common pathologies of systemic vascular disease. Blood vessels, together with nerves and lymphatic vessels, are wrapped in connective tissue membranes to form vascular nerve bundles. Differences in perivascular tissues, vascular nerve bundles, and intravascular structures result in altered vascular function. When imbalanced homeostasis is characterized by abnormal glucose and lipid metabolism, activation of the renin-angiotensin-aldosterone system (RAAS) and sympathetic nervous system (SNS) directly or indirectly causes widespread vascular damage throughout the body, leading to the development of panvascular complications of DM^15^ (Fig. 1). SNS dominates vasoconstriction and RAAS regulates of blood volume, vascular tone, and blood pressure.^16^ The vascular system in target organs is tightly regulated by surrounding tissues that regulate microvascular units through physical and signal transduction. As DM progresses, patients are more likely to develop various vascular complications and experience many pathological changes, such as endothelial dysfunction, AS, and microcirculatory disorders that interact with each other, consequently leading to the development of DPD.

Diabetic vasculopathy is classified as macroangiopathy and microangiopathy. Macroangiopathy includes AS of large and medium arteries (aorta, coronary, renal, basilar, and peripheral arteries), whereas microangiopathy includes endothelial damage to vessels between primary arterioles and venules, vascular basement membrane thickening, microthrombosis, platelet and red blood cell adhesion aggregation, and microcirculatory disorders. Due to differences in hemodynamics, vascular structure, and the affected cells, macro/microangiopathy present with different pathological manifestations. AS often occurs in sites of hemodynamic disturbances (preferably in elastic arteries). It manifests as macrophage foaming and EC lesions as well as mesangial SMC lesions. Microvessels are more hemodynamically stable, with a few cell layers, accompanying abundant plexus. On the other hand, differences in energy metabolic status, and organ-specific growth factors or cytokines in different target organs are also important components of the differences. Most target organs (heart, brain, peripheral vasculature) are affected by both diabetic macrovasculopathy and microvasculopathy; the retina and kidney are mainly affected by microvasculopathy (Fig. 1).

### DPDs

#### Diabetic heart disease (DHD)

DHD includes all kinds of cardiovascular diseases secondary to DM, including coronary artery disease (CAD) and cardiomyopathy. Cardiomyopathy and CAD are often treated separately as microvascular and macrovascular diseases, DM can increase the risk of both. They are often combined in clinical settings and ultimately lead to adverse outcomes and risk of death in patients with cardiovascular disease.^17^

Myocardial microvascular functional changes precede structural changes in patients with diabetes. These changes finally lead to extensive macrovascular AS.^18^ DM complicated with coronary atherosclerotic heart disease manifests as the segmental distribution of numerous vascular branches throughout the entire process, with obvious AS. Diabetic cardiomyopathy (DCM) refers to specific myocardial structural and functional abnormalities that occur in patients without CAD and cardiac risk factors such as hypertension, with heart failure (HF) as the main manifestation of chronic cardiovascular complications of DM. The main pathological features are left ventricular hypertrophy, myocardial fibrosis, cell necrosis, and other myocardial structural changes.^19,20^ Myocardial involvement in vasoactive metabolite secretion or neuromodulation causes changes in the coronary artery wall pressure or endothelial shear stress. Pericoronary adipose tissue can secrete adipokines and other vasoactive mediators and/or oxidative products that can directly alter the phenotypes of perivascular adipocytes.

Investigations of biomarkers of DHD and their applications have significantly progressed. Cardiovascular events as outcomes are more beneficial for the clinical application of biomarkers from the aspect of panvascular diseases. The severity of cardiovascular disease in patients with diabetes positively correlates with the ratio of oxidized low-density lipoprotein to low-density lipoprotein-cholesterol (Ox-LDL/LDL-C). This is considered a potential biomarker for the early identification and intervention of CAD in patients with diabetes.^21^ Osteopontin (OPN) is a multifunctional phosphorylated glycoprotein, that functions as an inflammatory cytokine and pro-atherosclerotic factor. High levels of OPN expression in the circulation and tissues are associated with cardiovascular complications in DM, and OPN is an independent predictor of cardiovascular disease in DM.^22^ Serum homocysteine levels are elevated in patients with T2DM and CAD and are closely related to the severity of coronary artery lesions.^23^ Plasma-free fatty acids also comprise an independent risk factor for CAD in patients with DM.^24^ Novel biomarkers are useful for providing insights into associations between DM and cardiovascular risk and developing treatment strategies for CAD associated with DM.

Coronary AS exists in diabetic patients, and the signal transduction of AS is similar in DPDs. Chronic inflammation, abnormal lipid metabolism, and secondary autoimmunity are the main mechanisms.^25^ ApoB-specific CD4 T cells have been identified in humans and mice, and treg can be induced with ApoB peptides.^26^ Hsp60/65 is the target antigen of autoimmune T Cells.^27^ Hyperglycemic states can further promote autoimmune responses. In addition to glycemic control and statins, monoclonal antibodies to proprotein convertase subtilisin/kexin type 9 (PCSK9), heat shock proteins60/65 (HSP60/65), and ApoB are expected to improve AS by targeting.^28^

**Fig. 2:**
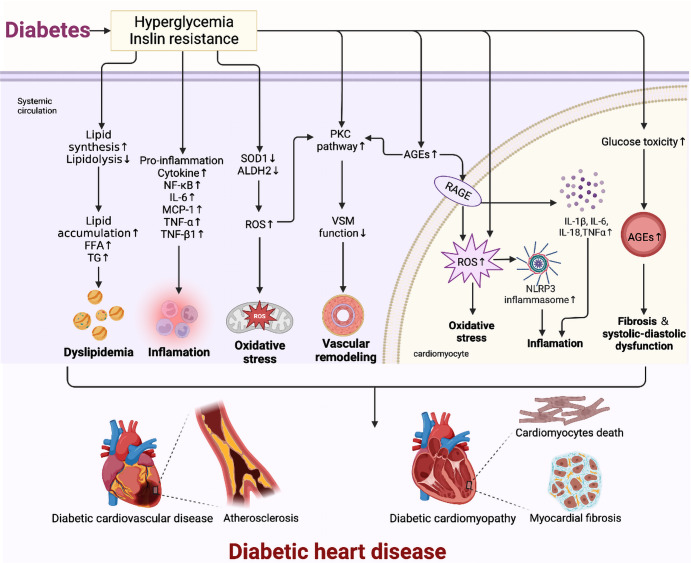
Pathology and molecular mechanisms of DHD. The mechanisms of diabetic heart disease are complex, including oxidative stress, inflammation, and altered metabolic pathways (advanced glycosylation end product (AGE) formation, PKC pathway), which intersect and work together to ultimately lead to myocardial remodeling and dysfunction

The mechanisms by which diabetes promotes cardiomyopathy have received attention, especially due to abnormal cardiac metabolism (Cardiac Metabolism), glycotoxicity and lipotoxicity, and abnormal mitochondrial function causing oxidative stress and inflammation.^29^ Unlike other target organs, the myocardium has high energy and oxygen requirements, and fatty acid oxidation (FAO) and aerobic oxidation of glucose are the main sources of energy for cardiac metabolism. Insulin resistance increases lipid synthesis in hepatocytes and lipolysis in adipocytes, leading to elevated circulating fatty acids and triglyceride levels. Lipid accumulation and fatty acids-induced lipotoxicity can affect myocardial FAO processes, promote endoplasmic reticulum (ER) stress, autophagy, and apoptosis, and cause ventricular remodeling.^20,30^ The most important metabolites of diabetic glycotoxin are advanced glycation end-products (AGEs) that are involved in the formation and evolution of DCM. These end-products bind cellular receptors of AGEs (RAGEs) that promote the production of reactive oxygen species (ROS), nuclear factor kappa-B (NF-κB), and pro-inflammatory cytokines such as interleukin (IL)-1β, IL-6, IL-18, tumor necrosis factor-alpha (TNF-α) that induce the intracellular production of abundant ROS, and initiate oxidative stress/inflammation cascade.^31,32^ AGEs/RAGE causing structural changes in the myocardium. Advanced glycation end-products also activate inflammatory signals through RAGEs on EC macrophages, and smooth muscle cells. This activation leads to increased ROS production and reduced nitric oxide synthesis, thus promoting the development of DCM.^33,34^ Hyperglycemia mediates a decrease in the expression of jund proto-oncogene subunit (JunD) and of free radical scavenger superoxide dismutase 1 and aldehyde dehydrogenase 2. It also mediates increased expression of inflammatory mediators such as NF-κB and Mcp-1, IL-6, and TNF-α that cause myocardial dysfunction and lead to the development of HF.^35^ Hyperglycemia promotes the increased expression of lncDACH1, which in turn promotes mitochondrial oxidative stress and apoptosis through increased ubiquitination-mediated degradation of NAD-dependent deacetylase sirtuin-3 (SIRT3), mitochondrial in mouse hearts, consequently aggravating DCM.^36^ Protein kinase C (PKC) is an effector in the G protein-coupled receptor system, and vascular SMC maintains vascular tone. PKC can be activated by excess ROS, AGEs, and diacylglycerol (DAG) to impair VSM function; this leads to vascular hyperresponsiveness and remodeling and accelerated development of DHD.^37,38^ Hyperglycemia triggers classical inflammatory pathways and oxidative stress.^39,40^ Hyperglycemia causes upregulates membrane cofactor protein-1 (MCP-1) and NLR family pyrin domain containing 3 inflammasome (NLPR3) expression, causing myocardial fibrosis and cardiac dysfunction, and exacerbated DCM development.^41^ Several molecular mechanisms synergistically act to impair the structural function of the heart and promote the development of DHD (Fig. 2).

Various interventions have conferred clinical benefits on patients with DHD (Table 1). Treatment of DHD should be comprehensive, with aggressive control of risk factors such as blood glucose, blood pressure, and lipids. Basic pharmacological therapy for CAD in patients with diabetes includes antiplatelet, cholesterol-lowering, anti-myocardial ischemia strategies, and RAAS inhibitors. Aggressive blood sugar control is necessary for treating DHD to avoid direct hyperglycemic damage. When treating T2DM complicated with cardiovascular disease, metformin should be combined with glucose-lowering drugs that have a proven cardiovascular benefit. When treating T2DM complicated with atherosclerotic cardiovascular disease, the preferred combination of metformin with either glucagon-like peptide-1 receptor agonist (GLP-1RA) or sodium/glucose cotransporter-2 inhibitors (SGLT-2i) can reduce cardiovascular events (Table 1). When treating T2DM complicated with HF, metformin should be combined with SGLT-2i, as this leads to a 39% reduction in the risk of HF hospitalization and a 46% reduction in the composite endpoint of HF hospitalization with all-cause death.^42^ Although GLP-1RA and dipeptidyl peptidase-4 inhibitors (DPP-4i) also confer some cardiovascular benefits, they do not offer a significant advantage for reducing the hospitalization of HF in patients with diabetes (Table 1).^43,44,45^

**Table 1:** Cardiovascular outcomes trials (including stroke) in diabetes mellitus (2013–2022)

Clinical trial	Clinical trials’ number	Year	Phase	Participants (*n*)	Intervention	Follow-up	Main outcome
**Dipeptidyl peptidase-4 (DPP-4) inhibitors**							
CARMELINA^475^	NCT01897532	2013–2016	3	6991	I: Iinagliptin C: placebo	2.2 years	Cardiovascular death, nonfatal myocardial infarction, nonfatal stroke
NA^476^	NCT01703208	2012–2016	3	4202	I: Omarigliptin C: placebo	1.84 years	Major adverse cardiovascular event, hospitalization for heart failure
SAVOR-TIMI 53^45^	NCT01107886	2010-2011	3	16492	I: Saxagliptin C: placebo	2.1 years	Composite of cardiovascular death, myocardial infarction, or ischemic stroke
TECOS^477^	NCT00790205	2008–2012	3	14671	I: Sitagliptin C: placebo	3 years	Composite of cardiovascular death, nonfatal myocardial infarction, nonfatal stroke, or hospitalization for unstable angina
**Glucagon-like peptide-1 receptor (GLP-1) agonists**							
REWIND^478^	NCT01394952	2011	3	9901	I: Dulaglutide C: placebo	5.4 years	Nonfatal myocardial infarction, nonfatal stroke, and death from cardiovascular
SUSTAIN-6^44^	NCT01720446	2013	3	3297	I: Semaglutide C: placebo	2.1 years	Cardiovascular death, nonfatal myocardial infarction, or nonfatal stroke
PIONEER 6^479^	NCT02692716	2017	3	3183	I: oral Semaglutide C: placebo	1.25years	Death from cardiovascular causes, nonfatal myocardial infarction, nonfatal stroke
EXSCEL^480^	NCT01144338	2010-2015	3	14752	I: Exenatide C: placebo	3.2 years	Death from cardiovascular causes, nonfatal myocardial infarction, nonfatal stroke
LEADER^481^	NCT01179048	2010–2012	3	9340	I: Liraglutide C: placebo	3.8 years	Death from cardiovascular causes, nonfatal myocardial infarction, nonfatal stroke
ELIXA^482^	NCT01147250	2010–2013	3	6991	I: Lixisenatide C: placebo	2.2 years	Death from cardiovascular causes, nonfatal myocardial infarction, nonfatal stroke, hospitalization for unstable angina
**Sodium-glucose cotransporter-2 (SGLT2) inhibitors**							
EMPA-REG OUTCOME^483^	NCT01131676	2015	3	7020	I: Empagliflozin C: placebo	3.1 years	MACE, cardiovascular, all-cause death, hospitalization for heart failure
CANVAS program^484,485^	NCT01032629 NCT01989754	2017	3	10142	I: Canagliflozin C: placebo	3.61 years	Cardiovascular death or hospitalized Heart failure
DAPA-HF^486^	NCT03619213	2018–2022	3	3131	I: Dapagliflozin C: placebo	2.3 years	Composite of worsening heart failure, cardiovascular death
Empa-HF^100^	NCT03485092	2018	3	150	I: Empagliflozin C: placebo	0.69 year	Left ventricular volumes
CREDENCE^487^	NCT02065791	2014–2017	3	4401	I: Canagliflozin C: placebo	2.62 years	Reduces the risk of kidney failure and cardiovascular events
DECLARE–TIMI 58^488^	NCT01730534	2013–2018	3	17160	I:Dapagliflozin C: placebo	4.2 years	MACE, composite of cardiovascular death, hospitalization for heart failure
EMPA-REG^489^	NCT01131676	2010–2013	3	7020	I:Empagliflozin C: placebo	3.1 years	Death from cardiovascular causes, nonfatal myocardial infarction, nonfatal stroke
**Others**							
SAVOR-TIMI 53^490^	NCT01107886	2010-2011	/	4894	I: Metformin C:Other antidiabetic drugs	2.1 years	Composite of cardiovascular death, myocardial infarction, or ischemic stroke
TOSCA.IT^491^	NCT00700856	2008–2014	3	3028	I: pioglitazone add on metformin C: sulfonylurea add on metformin	5 years	All-cause death, nonfatal myocardial infarction, nonfatal stroke, or urgent coronary revascularization
PROactive^492^	NCT00013208	2015	/	3606	I: Pioglitazone C: placebo	7.8 years	All-cause mortality, nonfatal myocardial infarction, stroke, cardiovascular mortality, cardiac intervention, et al
PROFIT-J^493^	UMIN000000846	2007–2011	3	481	I: Pioglitazone C: Other antidiabetic drugs	1.53/1.64 years	Composite of all-cause death, nonfatal cerebral infarction, and nonfatal myocardial infarction
DEVOTE^494^	NCT01959529	2013–2014	3	7637	I: Insulin Degludec C: Insulin Glargine	1.99 years	Death from cardiovascular causes, nonfatal myocardial infarction, nonfatal stroke
ORIGINALE^495,496^	NCT00069784	2012–2014	3	4718	C: Glargine I: Standard care	2.7 years	Death from cardiovascular causes or myocardial infarction or stroke and any of these three outcomes or hospitalization for heart failure or carotid, coronary, or peripheral revascularization
ACCORD^497^	NCT00000620	2001–2005	3	10251	I: intensive glycemic control C: standard glycemic control	3.7 years	Composite cardiovascular outcome, cardiovascular and total mortality, nonfatal myocardial infarction
Steno-2^498,499^	NCT00320008	1993–2006	3	160	I: intensive glycemic control C: standard glycemic control	13.3 years	Death from any cause Stroke
VADT^500^	NCT00032487	2000–2008	3	1791	I: intensive glycemic control C: standard glycemic control	5.6 years	Composite of major cardiovascular events
Look AHEAD^77^	NCT00017953	2001–2012	3	5145	I: intensive lifestyle intervention C: receive diabetes support and education	9.6 years	Composite cardiovascular outcome
AleCardio^501^	NCT01042769	2010–2012	3	7226	I: Aleglitazar C: placebo	2.5 years	Composite of cardiovascular mortality, nonfatal myocardial infarction, nonfatal stroke

*MACE* major adverse cardiovascular events, *NA* no official trial name

#### Diabetic encephalopathy (DE)

DM is significantly associated with an increased risk of several intracranial diseases, including cerebral macro- and microangiopathy.^46–48^ Broadly speaking, the intracranial complication of DM includes stroke, which can also manifest as depression, mild cognitive impairment (MCI), and dementia.^46,49–51^ Such vascular lesions can involve large carotid and vertebral arteries, small intracerebral perforating arteries, micro-arteries, capillaries, micro-venules, and small veins. They are also involved in disrupting the integrity of the blood-brain barrier (BBB) and neurodegeneration.^52,53^ The cerebral microvasculature facilitates intracranial nutrient delivery and waste removal, supports neuronal activity, maintains the interstitial environment, and reduces and stabilizes blood flow.^54^ Diabetic macroangiopathy and microangiopathy mutually promote each other, and the occlusion of macrovessels can cause chronic perfusion insufficiency in the brain and microvascular disorders.^55^ Microvascular function can affect collateral circulation in macrovessels^56,57^ and increase the risk of stroke^58^ as well as a poor prognosis.^59–61^ Neurons injury,^62^ Alzheimer’s like pathologies,^63^ and abnormal activity of neurotransmitter receptors^64^ are also closely associated with cerebrovascular lesions, and jointly cause brain function impairment in patients.

Elevated blood glucose is a risk factor for pathological changes in the brain and brain function impairment.^65,66^ Not only DM but also pre-DM can promote the development of dementia.^67^ However, the imaging changes do not correspond to the degree of cognitive impairment, and its mechanism deserves further exploration.^68^ Diabetic cerebral microangiopathy has multiple complex changes in images (cerebral atrophy, subcortical microinfarcts, cerebral white matter hyperintensity, lacunar infarction, perivascular space, and cerebral microhemorrhage),^69^ with diffuse adverse effects.^70,71^ Meanwhile, advances are being made in the measurement of cerebral microangiopathy, along with more precise MRI interpretations and artificial intelligence that have revealed more DM-induced cerebrovascular pathologies.^72^

**Fig. 3:**
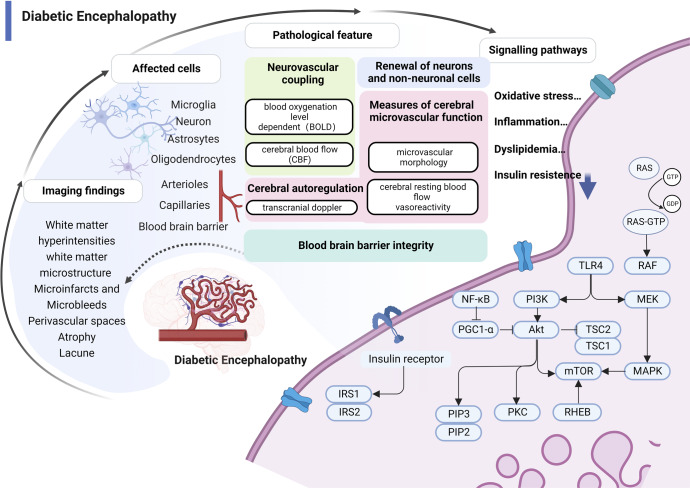
Pathology and molecular mechanisms of DE. (1) Structural brain changes from MRI studies in diabetes are the primary diagnostic basis of DE, including microinfarcts and microbleeds, perivascular spaces, white matter hyperintensities, white matter microstructure, lacunes, and atrophy; (2) Pathologies related to imaging findings include blood-brain barrier permeability, perfusion defects, hypoxia, and increased angiogenesis that can involve brain microvessels, multiple nerve cells, and the blood-brain barrier; (3) Microvascular dysfunction manifested by impaired neurovascular coupling and impaired neuronal function. Neurovascular coupling links transient local neural activity to subsequently increased blood flow; (4) The molecular pathways of hyperglycemic damage to brain microvasculature are closely related to oxidative stress, inflammation, abnormal lipid metabolism, and insulin resistance. RAS rat sarcoma protein, GTP guanosine triphosphate, GDP guanosine diphosphate, TLR4 Toll-like receptor 4, MEK mitogen-activated protein kinase, PI3K phosphoinositide 3-kinase, Akt protein kinase B, TSC tuberous sclerosis complex, MAPK mitogen-activated protein kinases, mTOR mammalian target of rapamycin, RHEB Ras homolog protein enriched in brain, PKC protein kinase C, PGC1-α PPAR-gamma co-activator-1 alpha, PIP2,3 phosphatidylinositol bisphosphate2, 3, IRS-1,2 insulin receptor substrate1, 2, NFκB nuclear factor kappa-B

Intracranial pathological changes of microangiopathy include endothelial dysfunction, platelet aggregation impairment, and increased inflammation.^73–75^ The expression of vascular endothelial growth factor (VEGF) and endothelin nitric oxide synthase (eNOS) are decreased in DE, which impairs cerebral artery endothelial function and results in decreased vascular autoregulatory response.^76^ Disorders of platelet aggregation and inflammation reduce cerebral blood flow and increase the risk of DE.^77^ Central nervous system is highly dependent on glucose for energy supply.^78–80^ Disturbance of carbohydrate metabolism can cause an intracranial energy metabolism imbalance and promote lesion development.^81,82^ Aldose reductase activity is significantly and systemically increased with hyperactivation of the sorbitol pathway in diabetes.^83^ This leads to insulin resistance, resulting in widespread oxidative stress and increased inflammatory cytokines. The insulin receptor signaling system plays an important role in maintaining normal brain and cognitive functions^84^ by regulating GLP-1 receptors,^84,85^ insulin receptor substrate (IRS) receptors.^86,87^ In other tissues, insulin activates the glucose transporter (GLUT) family of glucose transport proteins, but in the skull GLUT is directly regulated by glucose or cAMP. Activation of IRS or GLUT promotes glucose utilization in the Phosphatidylinositide 3-kinases (PI3K), protein kinase B (Akt), and β-arrestin/ extracellular regulated protein kinases (ERK) pathways. Insulin resistance causes chronic inflammation and increased oxidative stress, imbalanced energy metabolism.^88–92^ This leads to neuroinflammation and the apoptosis of pericytes and microglial cells,^88,89,92–96^ thus disrupting vascular endothelial tight junctions and causing damage to the BBB. The accumulation of AGEs also affects various cellular constituents of the BBB, resulting in increased BBB permeability and cognitive impairment.^97–99^ Vascular endothelial dysfunction further promotes the production of inflammatory mediators that disrupt the BBB,^59,63,64,100^ which exposes the brain parenchyma to potentially neurotoxic proteins.^101^ Classical inflammatory mediators such as IL-1β, IL-6, IL-10, TNF-α, vascular cell adhesion protein-1 (VCAM-1), and matrix metalloproteinases (MMP)-2 and -9 suggest vascular neuroinflammation. Intracranial-specific inflammatory signals, estrogen receptors promote increased expression of membrane Rα and ERβ in the hippocampus and promote hippocampal apoptosis;^102^ P38 activates mitogen-activated protein kinase (MAPK) pathway to promote neuronal cell death, and microglia activation.^103,104^ Hyperglycemia stimulates inflammatory signals and adaptive signals, accelerating ER stress and mitochondrial dysfunction.^105^ In addition, glutamate is a key excitatory neurotransmitter in the central nervous system. Glutamate receptors, including *N*-methyl-d-aspartic acid receptors (NMDA), may regulate neurogenesis and synaptic plasticity.^106,107^ Upregulation of NMDA receptors had beneficial effects on learning and memory in diabetic rats^108^ (Fig. 3).

The main therapeutic approaches target brain microvascular endothelium and the BBB, microvascular function, neuroinflammation, and antiplatelet agents.^109,110^ Maintaining normal blood glucose levels is essential, but no evidence supports intensive glucose-lowering regimens for patients with diabetes and cognitive impairment or dementia.^111–113^ Moreover, the risk of hypoglycemic events is associated with cognitive decline and increased risk of dementia.^81,82,114^ Although lowering glucose is generally ineffective for preventing stroke and cognitive impairment,^113^ some drugs do provide these benefits in addition to controlling glucose levels. Metformin, pioglitazone, and GLP-1 agonizts that can cross the BBB are of interest in the treatment of DE.^115^ Metformin might improve cognitive impairment associated with stroke or Alzheimer’s disease,^116,117^ and prevent dementia in persons aged <75 years more effectively than sulfonylurea hypoglycemic agents.^111^ Pioglitazone might reduce the risk of dementia by 47% in populations with diabetes.^48^ Pioglitazone and glitazones can activate peroxisome proliferator-activated receptor-γ (PPAR-γ),^118^ which can improve cell adhesion factors and inflammatory factors in brain cells. This receptor can also act on other tissues and regulate glucose metabolism and overall energy homeostasis.^119^ Abundant GLP-1α is expressed in the brain, and GLP-1 receptor agonizts have a good safety profile, neuroprotective effects, and can improve cognitive impairment. The new glucose-lowering agent SGLT-2i has cardio-renal protective effects, but it might increase the risk of stroke, which makes its use controversial.^120–122^ Furthermore, the mechanism of SGLT-2i actions is unknown. Meanwhile, new benefits have been confirmed for some traditional hypoglycemic agents, such as glibenclamide, which can reduce hemispheric edema after stroke when intravenously injected.^123,124^ Among non-hypoglycemic drugs, phosphodiesterase type III inhibitor Cilostazole^125^ improves oxidative stress and regulates cerebrovascular damage. NMDA receptor agonizts, neurotrophic factors, and mitochondrial function modifiers are also under development.^126^

Imaging modalities such as MRI are significantly more important than serum markers for identifying encephalopathy compared with other diabetic vascular lesions. However, current imaging protocols for diagnosing DE are not sufficiently specific. The correlation between imaging and corresponding molecular mechanisms is not yet clear. To overcome this limitation, large clinical trials targeted other vascular lesions and glycaemic control need to be conducted, to identify the population with diabetes that is at high risk of developing cerebrovascular lesions.^82^

#### Diabetic kidney disease (DKD)

**Fig. 4:**
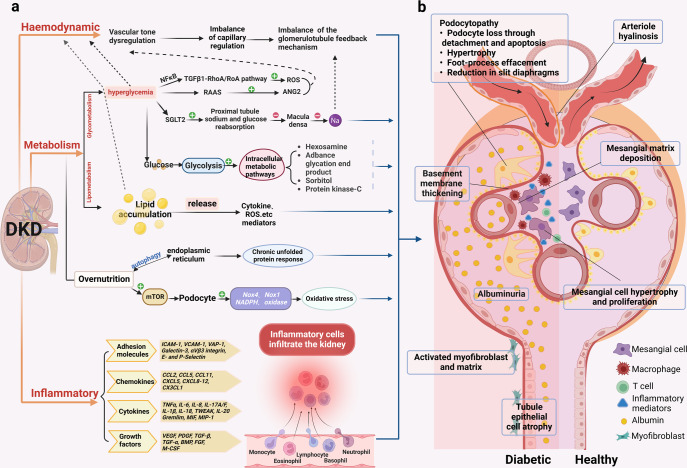
Pathology of the glomerulus and tubules in DKD. **a** The classical pathological mechanisms of DKD. It mainly includes hemodynamic, metabolic disturbances, and inflammation, which often interact with each other. (1). Hemodynamic disturbances lead to dysregulation of tubulobulbar feedback balance. (2). Metabolic disorders are crucial to the pathogenesis of DKD. Hyperglycemia affects pathways such as TGFβ1-RhoA/Roa pathway, RAAS, proximal tubular sodium and glucose reabsorption, and intracellular metabolism; abnormal lipid metabolism can affect the release of mediators such as cytokines and ROS; in the presence of nutrient overload in the organism, endoplasmic reticulum autophagy leads to a chronic unfolded protein response, and mTOR also disturbs the podocytes leading to oxidative stress. (3). Inflammation promotes the release of inflammatory mediators such as adhesion molecules, chemokines, cytokines, and growth factors, causing renal infiltration of inflammatory cells. **b** Schematic representation of the pathological damage of DKD. Differences in structural changes of glomeruli and tubules in the diabetic setting and in the healthy state. Diabetic glomerulopathy is characterized by arterial hyalinization, thylakoid stromal deposition, basement membrane thickening, glomerular thylakoid cell hypertrophy and proliferation, podocytosis, proteinuria, tubular epithelial atrophy, activated myofibroblasts, and stromal accumulation. NFκB nuclear factor kappa-B, TGFβ transforming growth factor-β, ROS reactive oxygen species, RAAS renin-angiotensin-aldosterone system, ANG2 angiotensin II, SGLT2 sodium-dependent glucose transporters 2, mTOR mammalian target of rapamycin, NADPH nicotinamide adenine dinucleotide phosphate, NOX NADPH oxidase, ICAM-1 intercellular cell adhesion molecule-1, VCAM-1 vascular cell adhesion molecule-1, VAP-1 vascular adhesion protein-1, CCL CC chemokine ligand, CXCL C-X-C motif chemokine ligand, TNF tumor necrosis factor, IL interleukin, TWEAK tumor necrosis factor-like weak inducer of apoptosis, MIF macrophage migration inhibitory factor, MIP-1 macrophage inflammatory protein-1, VEGF vascular endothelial growth factor, PDGF platelet-derived growth factor, BMP bone morphogenetic protein, FGF fibroblast growth factor, M-CSF macrophage colony-stimulating factor

Between 5 and 40% of patients with diabetes eventually develop diabetic kidney disease (DKD),^127^ and the number of those with chronic DKD is increasing by 2.62 million annually, with chronic DKD being the leading cause of end-stage renal disease.^128^ DKD is a microvascular complication characterized by glomerular hypertrophy, basement membrane thickening, and damage.^129^ The complex renal vascular system, including the renal arteries and their branches, as well as glomerular and peritubular capillary networks, form the basis for maintaining normal renal function. The mechanism through which DM causes kidney damage is complex and might involve hemodynamic, metabolic, and inflammatory pathways and targets^130,131^ (Fig. 4). A hyperglycemic environment induces hypertension, which increases the disturbed renal perfusion pressure and indirectly causes microvascular damage in renal arteries, glomerular and tubulointerstitial capillaries.^132–134^ AS causes thickening of renal artery walls and lumen narrowing.^135,136^ Upregulated SGLT expression promotes glucose uptake, which affects the tubular-glomerular feedback mechanism, leading to glomerular hypertension.^137,138^ Plasma levels of kallikrein, thrombin, and coagulation factors are elevated in hyperglycemic states. A chronically activated coagulation system is closely associated with a vascular injury in patients with DKD.^139^

The most prevalent diagnostic markers for DKD are the estimated glomerular filtration rate (eGFR) and proteinuria calculated based on serum creatinine or cystatin C.^140^ However, tissue damage is often irreversible by the time a diagnosis is confirmed, and new biomarkers are needed to diagnose DKD earlier and to stratify risk factors. Current new biomarkers mainly target diagnoses of inflammation, endothelial damage, fibrosis, endothelial dysfunction, and kidney damage,^141^ including TNF-α receptor,^142^ intercellular adhesion molecule-1,^143^ endostatin,^144^ copeptin,^145^ kidney injury molecule-1,^146,147^ monocyte chemoattractant protein-1,^148^ and neutrophil gelatinase-associated lipocalins.^149^ Extracellular vesicles have recently attracted interest,^150^ because they are essentially exosomes and particles^151^ that transport miRNA,^152^ mRNA,^153,154^ and proteins,^155^ and might serve as early biomarkers of DKD. In addition to individual biomarkers, other approaches such as proteomics,^156^ genomics,^157^ and metabolomics^158^ all play roles in screening, especially in studies of chronic kidney disease (CKD)^159^ which can predict the development of proteinuria.^160^ Despite many studies of novel biomarkers, only eGFR and proteinuria are routinely applied to clinically diagnose DKD, and the specificity and accuracy of these awaits clarification in future controlled clinical trials with large samples.

In addition, imbalanced pro- and anti-angiogenic factors can disrupt the vascular network in DKD. Hyperglycemia increases glomerular capillary pressure through a RAAS-mediated increase in angiotensin II.^161,162^ Hyperglycemia-mediated changes in glomerular capillary autoregulation can cause endothelial dysfunction and inflammation by increasing transforming growth factorβ-1 (TGFβ-1) mediated ROS that dilates glomerular afferent and efferent arterioles.^163^ Lipid metabolism is often abnormal in patients with diabetes, and this can also contribute to glomerular and tubulointerstitial vascular injury through mediators such as cytokines, ROS, and hemodynamic changes.^164,165^ Hypoxia in renal tissues due to reduced density of tubulointerstitial capillaries is also a major cause of renal disease progression.^166,167^ Although scant vascular endothelial growth factor A (VEGFA) is expressed in renal tubular capillaries, the absence of specific VEGFA expression leads to a significant decrease in peritubular capillary density in mice.^168^ However, overexpression causes dilation of peritubular capillaries.^169^

Inflammatory mediators (chemokines, cytokines, and adhesion molecules)^170,171^ are often released due to hyperglycemia and hemodynamic abnormalities, which lead to nephron damage through ultrafiltration, mechanical stress, oxidative stress, glycocalyx dysfunction, and endothelial activation.^172,173^ These mediators cause renal microvascular dilation or altered permeability through thylakoid proliferation, podocyte or tubular injury,^174^ and inflammatory cell infiltration.^175^ In addition, oxidative stress is closely associated with inflammation and causes endothelial dysfunction by activating NF-κB^176,177^ and adapter protein complex-1^178,179^ to induce pro-inflammatory factors and, in turn, mediate the inflammatory response, thus inducing renal fibrosis.^180^ Therefore, the main causes of vasculopathy in DKD are inflammation, hemodynamic, and metabolic disorders. Lesions are mostly concentrated on the glomerular and tubulointerstitial microvasculature and also in other vessels such as renal arteries, and glomerular afferent and efferent arterioles. The pathogenesis and therapeutic strategies need further exploration.

The current strategies for treating DKD mainly comprise controlling blood sugar and blood pressure and blocking the RAAS^140^ (Table 2). After controlling various risk factors such as hyperglycemia, hyperlipidemia, hypertension, and uric acid,^181,182^ the risk of DKD is reduced, but the vascular disease continues to progress. Blood pressure control and fenofibrate can increase the risk of renal adverse events such as decreased eGFR, suggesting a need to explore more effective treatment modalities.^183,184^ Novel hypoglycemic agents might protect the kidney through combined actions against hyperglycemia, hypertension, lipotoxicity,^185^ abnormal tubuloglomerular feedback,^186^ hypoxia,^187^ endothelial dysfunction, and renal fibrosis.^188^ The SGLT-2i dapagliflozin exerts renoprotective effects, possibly by affecting the hemodynamics of patients mainly through post-glomerular vasodilation to normalize the eGFR.^189^ In addition to stimulating insulin secretion from pancreatic β-cells to control blood glucose, incretin might also bind to GLP-1 receptors to inhibit endothelial damage and thus exert positive effects.^190^ The large FIDELIO-DKD clinical trial found that finerenone, a novel nonsteroidal mineralocorticoid receptor antagonist combined with a RAAS inhibitor, reduced the risk of cardiac and renal outcomes while reducing the incidence of hyperkalemia.^191^ MiRNAs play an important role in maintaining optimal vascular homeostasis and regulating microvasculature disorders.^192^ The miR-132 inhibitor CDR132L improves HF and affects cardiac fibrosis biomarkers^193^ and might also have a therapeutic effect on renal fibrosis. Anticoagulants might hamper the progression of DKD,^194^ but the clinical application requires further validation and tests, as some anticoagulants such as vorapaxar might increase bleeding risk (Table 2).^195^

**Table 2:** Renal outcomes trials in diabetes mellitus

Clinical trials	Clinical trials’ number	Year	Phase	Paticipants (*n*)	Intervention	Follow-up	Main outcome
**Dipeptidyl peptidase-4 (DPP-4) inhibitors**							
CARMELINA^502^	NCT01897532	2013–2018	4	6991	I: Linagliptin C: Placebo	2.2 years	Reduced proteinuria, control blood glucose.
**Glucagon-like peptide-1 receptor (GLP-1) agonizts**							
REWIND^503^	NCT01394952	2011–2018	3	9901	I: Dulaglutide C: placebo	5.4 years	Reduced compound renal outcome.
PIONEER 5^504^	NCT02827708	2016–2018	3	424	I: Semaglutide C: Placebo	0.54 years	Effective in patients with type 2 diabetes and moderate renal impairment, but with higher adverse events.
BETENT-4^505^	NCT03730662	2018–2021	3	2002	I: Tirzepatide C: Insulin Glargine	2 years	Slowed down eGFR decline rate, reduced UACR (urinary albumin creatinine ratio).
**Sodium-glucose cotransporter-2 (SGLT2) inhibitors**							
DAPA-CKD^506^	NCT03036150	2017–2020	3	4304	I: Dapagliflozin C: Placebo	3.2 years	Reduced the risk of GFR and major renal and cardiovascular adverse events in diabetic and non-diabetic patients with chronic kidney disease.
CREDENCE^487^	NCT02065791	2014–2018	3	4401	I: Canagliflozin C: Placebo	2.62 years	Reduced the risk of kidney failure and cardiovascular events.
SCORED^507^	NCT03315143	2017–2020	3	10584	I: Sotagliflozin C: Placebo	1.3 years	Reduced risk of cardiovascular-related hospitalization and death from diabetes and CKD, but associated with adverse events.
VERTIS CV^508^	NCT01986881	2013–2019	3	8223	I: Ertugliflozin C: Placebo	3.5 years	Ertugliflozin reduced the risk of composite renal end points and was associated with reduced eGFR and UACR.
**Mineralcorticoid receptor antagonists**							
FIDELIO-DKD^191^	NCT02540993	2015–2021	3	5734	I: Finerenone C: Placebo	2.6 years	Reduced the risk of the cardio-renal outcome.
PRIORITY^509^	NCT02040441	2014–2018	2/3	209	I: Spironolactone C: Placebo Standard care	2.5 years	Can’t prevent disease progression of high-risk patients with DKD.
SONAR^510^	NCT01858532	2013–2018	3	2648	I: Atrasentan C: Placebo Standard care	4.4 years	Reduced the risk of renal events in patients with diabetes and CKD.
**Others**							
CKD-FIX^181^	ACTRN12611000791932	2014–2016	3	369	I: Allopurinol C: Placebo Standard care	2.17 years	Decreased serum urate but did not affect the renal outcome and did not alleviate the decline in eGFR.
ALBUM^197^	NCT02358096	2015–2017	2	125	I:ASP8232 C: Placebo	2 years	Reduced albuminuria in DKD patients, safe and well tolerated.
NA^196^	NCT01683409	2012–2017	2	130	I: Baricitinib C: Placebo	0.46 years	Reduced albuminuria.
NA	NCT03804879	2018–2021	2	83	I: Nidufexor C: Placebo	0.54 years	UACR and 24-hour urinary albumin were decreased in DKD patients.
NA^201^	NCT03016832	2017–2021	1	413	I: HuangKui capsule C: Irbesartan tablets	0.46 years	The combination of Huangkui capsule and irbesartan had the best effect on reducing ACR in DKD patients.

*NA* no official trial name

Some pathways associated with kidney injury have emerged as novel targets for treating DKD. For example, baricitinib is an oral small-molecule inhibitor that selectively inhibits the Janus kinase (JAK) protein tyrosine kinase family members JAK1 and JAK2,^196^ which inhibits the JAK-mediated inflammatory pathways and reduces proteinuria. Small-molecule inhibitors associated with kidney damage, such as the vascular adhesion protein-1 inhibitor ASP8232, reduce proteinuria through local effects on glomeruli and podocytes, offering potential multi-target interventions.^197^ Another promising therapeutic target is gut flora, as the renal disease causes the dysbiosis of various gut microbes. Inhibiting phenyl sulfate (a metabolite derived from gut microbiota) reduces proteinuria in mice with DKD.^198^ Supplementing patients with diabetes who are on hemodialysis with probiotics improves glucose and lipid metabolism, as well as biomarkers of inflammation and oxidative stress.^199^ Orally administered *Faecalibacterium* exerts both anti-inflammatory and renoprotective effects on patients with CKD through butyrate-mediated G protein-coupled receptor-43 signaling.^200^ Moreover, traditional Chinese medicine has also achieved considerable progress in DKD treatment, as a combination of Huangkui capsules and irbesartan was found to be more effective than either of the medications alone in reducing albumin-to-creatinine ratio in patients with DKD,^201^ suggesting the potential role of Chinese medicine in future DKD therapy.

Renal microvasculature is an important target of DPD. Intensive management of DM, including controlling blood sugar and blood pressure and blocking the RAAS, will reduce the incidence of CKD and delay its progression. Current and future health resource requirements for DKD treatment are difficult to estimate. Thus innovative therapeutic strategies are needed to prevent, block, treat, and reverse DKD.

#### Diabetic retinopathy (DR)

DR is one of the most common microvascular complications of DM.^202,203^ Pathological changes of DR start with the loss of retinal neuronal. The series of events include early loss of neurovascular coupling, retinal neurodegeneration, and subsequent gliosis, finally leading to retinal vasculopathy. Microvasculopathy in the DR retina are manifested as loss of retinal capillary epithelial cells, decreased capillary elasticity, and increased vascular permeability, exudation, local inflammation, and growth factors promoting neovascularization.^204,205^ DR is clinically classified as non-proliferative (NPDR) or proliferative (PDR) in the absence or presence of retinal neovascularization, respectively. The NPDR type progresses to PDR and eventually develops into macular edema, with the latter being an important cause of vision loss or blindness in patients with diabetes.^206,207^ Such patients must undergo regular fundus examinations to detect vision-threatening stages of DR, such as PDR and diabetic macular edema, as early as possible to treat them before vision loss becomes irreversible.

Delayed diagnosis and treatment are the most common causes of visual impairment in patients with diabetes. Therefore, the early detection and prevention of lesions in DR is the key to stop DR progression. Biomarker-related biomic and artificial intelligence (AI) investigations will play increasingly important roles in the risk assessment, early diagnosis, and treatment of the disease. Homocysteine levels are significantly high in the serum of patients with diabetes and should become a screening and diagnostic indicator of DR, as its prevention and treatment can be targeted by increasing homocysteine clearance.^208^ The main factor controlling neovascularization is VEGF, levels of which increase in vitreous and tear fluids from patients with DR and correlate positively with DR severity. Retinol-binding protein 3 (RBP3) is a retinol transporter protein secreted by photoreceptors, and high levels of RBP3 in the vitreous body of patients with diabetes slow DR progression.^209^ Elevated RBP3 expression can alleviate hyperglycemia-induced DR by inhibiting glucose uptake by glucose transporter protein-1 and reducing the expression of inflammatory cytokines and VEGF.^210^ Therefore, RBP3 could serve as a biomarker and therapeutic strategy in preventing the progression of DR. MiRNAs are involved in retinal neovascularization and the inflammatory response in DR. The relative expression of serum miRNAs was measured in 80 patients with T2DM comprising an NPDR group with normal, mild, moderate, or severe symptoms and a PDR group. The results showed that the relative expression of serum miR-146a and miR-21 increased, whereas that of miR-34a decreased with worsening DR severity.^211^ miR-125a-5p significantly attenuated vascular leakage in DR.^212^ These findings suggested that miR-146a, miR-21, miR-34a, and miR-125a-5p could serve as promising biomarkers for DR.^211^ AI has recently become a research hotspot in auxiliary medical diagnosis. Ophthalmic AI integrates imaging databases with deep learning (DL) technology to automatically measure and analyze the characteristic biological structures of the eyes of patients with DR to assist with diagnosis. We found different features of Hematoxylin-eosin-stained retinal sections from diabetic mouse models based on changes in nerve fiber layers and ganglion cells during the early stage of the disease. We then identified these features using image recognition and DL, and consequently identified changes in ganglion cells and the nerve fiber layer that could be applied to the early quantitative diagnosis of DR.^213^ Another AI-based study by our team quantified the pathological changes of retinal neurons and synapses in mice with diabetes induced by monosodium glutamate (MSG). We found that MSG-induced DR was closely associated with neurotransmitter abnormalities and had important features of retinal neurodegeneration, providing an effective animal model and technique for quantifying retinal neuron pathology.^214^

**Fig. 5:**
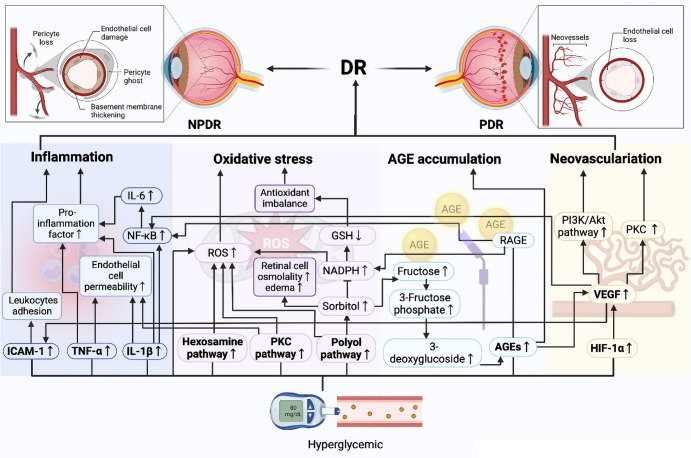
Pathology and molecular mechanisms of DR. Multiple mechanisms are involved in the pathogenesis of DR. Hyperglycemia can promote oxidative stress through the polyol pathway, accumulation of advanced glycosylation end-products (AGEs), the protein kinase C (PKC) pathway, and the hexosamine pathway, and exacerbate inflammation and abnormal angiogenesis by stimulating the secretion of inflammatory factors and vascular endothelial growth factor, inducing retinal dysfunction until vision loss

A thorough knowledge of the mechanism of DR is essential for its prevention and treatment. Hyperglycemia can lead to inflammation, oxidative stress, and increased glycosylation product and VEGF contents during the late stages. These can increase retinal permeability and alter retinal hemodynamics, leading to retinal leakage and the development of DR. Serine racemase (SR) promotes the formation of d-serine, which activates NMDA receptors and has multiple effects on neuron.^215^ Overexpression of SR in diabetic retinopathy leads to retinal neurodegeneration. Hyperglycemia subsequently leads to vascular EC damage, and in turn, leukocyte aggregation/adhesion to vessel walls.^216^ Leukocyte adhesion and aggregation activate a massive amount of neutrophils that adhere to EC and form a reticular network and aggravate tissue hypoxia, causing vascular remodeling and neovascularization.^217^ The main protein that mediates intercellular adhesion is intercellular cell adhesion molecule-1 (ICAM-1).^218^ Under inflammation, ICAM-1 is abundantly expressed in retinal EC, where it binds to receptors. This induces leukocytes to penetrate the endothelium and become adherent, indicating that ICAM-1 is an important mediator of the inflammatory response in DR.^219,220^ The inflammatory response of the retina in DR involves the production and release of various inflammatory factors.^221^ In particular, the release of massive amounts of IL-1β can lead to the apoptosis of retinal pigment epithelial cells, which damages the integrity of photoreceptors. IL-1β activates NF-κB and oxidative stress, leading to the apoptosis of capillary EC and increasing EC permeability.^222^ IL-1β also promotes IL-6 secretion and induces capillary angiogenesis by activating the NF-κB pathway and p38/MAPK.^223^ The pro-inflammatory factor TNF-α can cause EC damage and increase EC permeability, resulting in vascular leakage.^224^ Inflammatory factors induce each other *via* cascade amplification, mediating the inflammatory response and exacerbating DR. Oxidative stress leads to nerve, vascular, and retinal tissue damage and, in turn, the development of DR.^225,226^ Chronic hyperglycemia causes oxidative stress mainly through PKC, polyol, hexosamine, and AGEs formation pathways.^225^ Hyperglycemia can regulate vascular cell permeability, the extracellular matrix, cell growth, neovascularization, cytokine response, and leukocyte adhesion through the diacylglycerol-PKC pathway, leading to structural and functional changes of the retinal vasculature.^227,228^ Activation of the polyol pathway produces oxidative stress, which increases the consumption of reduced coenzyme II (NADPH) oxidase through the production of sorbitol by aldose reductase (AR), thus affecting the production of the antioxidant reduced glutathione and causing an oxidative-antioxidative imbalance.^229–231^ The production and accumulation of sorbitol under hyperglycemic conditions increases retinal osmotic pressure, cell edema, metabolic disorders, and microvascular damage,^232,233^ consequently aggravating DR. Advanced glycation end-products promote NF-kB activation by interacting with the cellular RAGEs on the cell surface.^234^ This leads to retinal pericyte apoptosis and elevated expression of VEGF, inflammatory cytokines, and adhesion molecules.^235^ Inhibition of ACEs can improve hyperglycemia-induced blood-retinal barrier leakage and reduce retinal EC proliferation, migration, and neovascularization,^236^ thus alleviating DR. The main hallmark of PDR is neovascularization. The most important inflammatory factor that stimulates neovascularization and causes vascular leakage is VEGF.^209^ Hypoxia-inducible factor-1α is activated under hyperglycemic and hypoxic conditions, which leads to increased secretion of VEGF, and overexpressed VEGF, in turn promotes neovascularization through activation of the PI3K/Akt,^237–239^ PKC,^240,241^ and NF-κB^242,243^ signal pathways. The expression of ICAM and nitric oxide synthase induced by VEGF promotes leukocyte adhesion and causes changes in vascular permeability and pathological neovascularization.^244–246^ The pathogenesis of DR is complex, with numerous factors that synergistically interact with each other during the development and progression of DR (Fig. 5).

DR can be treated mainly by laser photocoagulation and vitreous injections of antibodies.^247^ Retinal laser photocoagulation can prevent further vision deterioration, but cannot restore damaged vision. Intravitreally injected ranibizumab and bevacizumab reduce the recurrence of active neovascularization and risk of retinal detachment. However, frequent intraocular administrations due to a short half-life increase the risk of retinitis, retinal obstruction, and patient pain and might facilitate the development of drug resistance.^248,249^ Intraocular glucocorticoid injection is often used to treat persistent and refractory diabetic macular edema, which improves vision but increases the incidence of cataracts and glaucoma.^250^ Drugs such as ranibizumab, aflibercept, and fenofibrate have been included in clinical trials to evaluate their effectiveness and safety (Table 3), thus providing new ideas for treating DR.

**Table 3:** Interventional trials in diabetic retinopathy

Clinical trials	Clinical trials’ number	Year	Phase	Participants (*n*)	Intervention	Follow-up	Main outcome	Adverse events
**Sodium-glucose cotransporter-2 (SGLT2) inhibitors**								
Dapagliflozin^511^	NCT02919345 (Completed)	2017	/	97	I: Dapagliflozin C: glibenclamide	12 weeks	Central retinal thickness	Not reported
Dapagliflozin plus another oral hypoglycemic agent	NCT05310916 (Ongoing)	2022	3	60	I:Dapagliflozin 10 mg Tab plus another oral hypoglycemic agent C:Two oral hypoglycemic agents other than dapagliflozin	12 weeks	Severity of retinopathy	/
**Others**								
Faricimab^512^	NCT03622580 (Completed)	2018	3	1891	I:faricimab C:faricimab per personalized treatment interval or aflibercept	1 year	Mean change in best-corrected visual acuity	Intraocular inflammation
Brolucizumab^513^	NCT03321513 (Completed)	2017	3a	270 (312 eyes)	I:Brolucizumab C: aflibercept	2 years	Mean best-corrected visual acuity; Retinal central subfield thickness and visual acuity	Retinal vasculitis and retinal vascular occlusion
Abicipar^514^	NCT02462928 NCT02462486 (Completed)	2015	3	1888	I: Abicipar C: ranibizumab	52 weeks	Stable vision, best-corrected visual acuity, central retinal thickness	Intraocular inflammation
Fenofibrate^515^	NCT01927315 (Completed)	2013	4	41	I: Fenofibrate C: placebo	12 weeks	Change in the levels of circulating hematopoietic stem/progenitor cells	Ischaemic stroke related to pre-existing conditions
PDS implant pre-filled with ranibizumab	NCT04503551 (Ongoing)	2020	3	174	I: PDS implant pre-filled with ranibizumab C: Intravitreal ranibizumab	52 weeks	Early treatment diabetic retinopathy study, diabetic retinopathy severity scale	/
OPL-0401	NCT05393284 (Ongoing)	2022	2	120	I: OPL-0401 C: Placebo	24 weeks	Improvement in diabetic retinopathy severity scale	/
Calcium Dobesilate	NCT04283162 (Ongoing)	2020	4	1200	I:Calcium dobesilate + conventional treatment C:conventional treatment	12 months	The rate of the progression of diabetic retinopathy	/
Sinemet CR	NCT05132660 (Ongoing)	2022	1	244	I:Sinemet CR C:placebo	24 months	Electroretinogram	/
Aflibercept	NCT04708145 (Ongoing)	2021	4	150	Eyes without panretinal photocoagulation (PRP) and eyes with PRP, Drug: Aflibercept Injection	112 weeks	Improvement in diabetic retinopathy severity scale	/

#### Diabetic peripheral vasculopathy (DPVD)

DPVD is often overlooked, yet it is one of the most important and common vascular complications in patients with T2DM.^251^ In such patients, DPVD increases the risk of not only coronary atherosclerotic events,^252^ but also major adverse limb events such as amputation.^253^ DPVD can manifest as diabetic foot syndrome and peripheral arterial disease (PAD), which seriously affect the quality of life of patients with diabetes. Peripheral arterial disease is traditionally considered to be dominated by large artery AS.^254^ In fact, PAD is often accompanied by local and systemic microangiopathy.^255,256^ Multivessel endothelial dysfunction can manifest as microangiopathy (either exclusively or with other diseases), such as capillary basement membrane thickening, endothelial hyperplasia, oxygen tension reduction, and hypoxia, affecting peripheral nerve function.^257^ Pre-DM can affect blood vessels and accompanying nerves.^258,259^ The chronic course of DM might have further adverse effects under poor glycemic control.^260,261^ An increase in postprandial glucose plays an important role in the development of peripheral vascular disease in DM.^262^

The pathogenesis of DPVD overlaps with that of other AS and microvascular endothelial injuries. IL-6, high-sensitivity C-reactive protein, lipoprotein-associated phospholipase A2, and high-molecular-weight lipocalin biomarkers serve as indicators of the risk of cardiovascular disease and peripheral vascular disease.^263–267^ Specific markers for DPVD are unknown, but many biomarkers of vascular injury are available.^268,269^ For example, circulating levels of ICAM and sE-selectin indicate EC activation and vascular inflammation, and thus have potential as diagnostic markers of DPVD.^270,271^

The molecular mechanisms of AS in DPVD can be found in the section on coronary AS. Microvasculopathy in DPVD is highly concomitant with neuropathy, as microvessels form a neurovascular network with accompanying nerves.^272^ Neurons and Schwann cells are highly susceptible to hyperglycemia.^273,274^ Energy and inflammation, oxidative stress,^275^ insulin resistance,^276^ AGEs,^277^ nerve growth factors,^278^ activation of the polyol pathway,^279^ and activation of the hexosamine and PKC pathways^280^ are core pathological factors and processes similar to those of other DM vascular complications.^281,282^ Glucose and fatty acid metabolism,^283^ neural metabolism,^284^ and exosome regulation have recently attracted attention.^285,286^ Much more is known about peripheral neuropathy than about DPVD. Glucose overload and high fatty acid metabolism lead to decreased ATP production, excessive ROS formation, and impaired mitochondrial function, which further increases oxidative stress, leading to the formation of AGEs from the glycosylation of various proteins. The vicious cycle of these events further promotes ROS formation and ER stress, resulting in DNA damage and apoptosis of various cells. Abnormal neurometabolism in DM manifests as changes in sphingolipid metabolism, wherein sphingolipids are biologically active and important structural components of plasma cell membranes and are important signaling molecules. Abnormal sphingolipid metabolism causes neurotoxicity in patients with hyperglycemia.^287^ All of these pathways eventually manifest as increased pro-inflammatory factors that further induce AGEs production, leading to oxidative stress and endothelial dysfunction.^288^ These interactive processes simultaneously place EC and neurons in a state of oxidative stress and inflammation.

The endpoint of DPVD is amputation, the occurrence of which is closely associated with infection and trauma. Therefore, care and lifestyle changes play an important role in its treatment, which greatly differs from the preventive measures for other vascular pathologies. Normal or near-normal glycemic control is a primary therapeutic goal. Intensified hypoglycemic therapy reduces the incidence of peripheral neuropathy in patients with T1DM but has a little additional benefit for those with T2DM.^289,290^ Systemic antioxidant and anti-inflammatory therapy might also provide some benefits.^291^ New glucose-lowering drugs can reduce blood glucose without increasing the risk of amputation. In a subgroup of patients with T2DM combined with PAD, empagliflozin reduced rates of mortality, hospitalization for HF, and the progression of kidney disease.^252^ Patients treated with the new glucose-lowering drugs SGLT-2i, GLP-1RA, and DPP-4i have low risks of amputation with a good safety profile.^292^ Some glucose-lowering agents have conferred advantages for patients with DPVD and other multivessel diseases. The results of the LEADER trial suggested that liraglutide could be used in diabetic multivessel diseases.^293^ These treatment options could improve the overall quality of life of patients.

### Temporal progression of DPDs

The time of onset of panvascular complications in patients with diabetes is closely associated with patient age,^294^ race,^295^ genetic background,^296^ DM staging,^297^ treatment regimen,^298,299^ and level of glycemic control.^300,301^ The natural course of DPDs is not completely clear, but diabetic microangiopathy generally precedes diabetic macroangiopathy.^302^ Clinical trials of patients with insulin-dependent DM have found that DR develops within the first 2 years of DM in conventionally treated patients, and that by the fifth year, 25–40% of these patients develop retinal, renal, and peripheral microangiopathy.^303^ A Chinese cohort study has shown that >20% of patients develop moderate retinopathy and >40% develop mild proteinuria within 7 years after the onset of DM.^304^ According to the ADVANCED study, the incidence of macroangiopathy at the fifth year of DM does not exceed 10%.^305^ Complications with panvascular disease appear earlier and more frequently in patients with T2DM, than in those with T1DM.^306,307^ After adjusting for age and the duration of DM, the risk of peripheral neuropathy is found to be significantly higher in patients with T2DM than in those with T1DM.^306^ The median elapsed time to the onset of microproteinuria is also significantly shorter in adolescents with T2DM than in those with T1DM (1.3 vs. 6.8 years).^307^

**Fig. 6:**
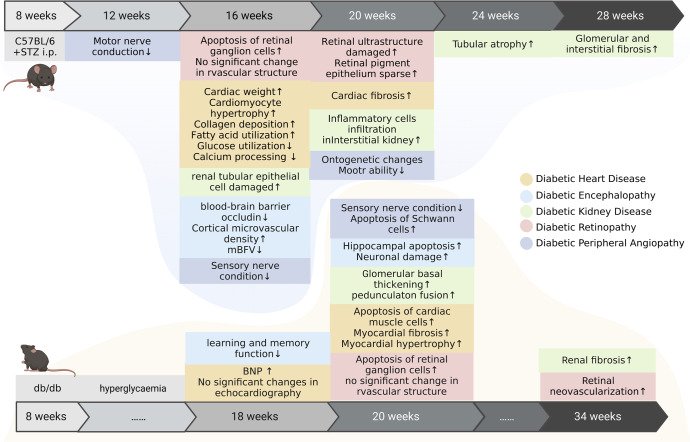
The natural course of diabetic vasculopathy in mice. Streptozotocin induced in C57BL/6 mice simulate type 1 diabetes in human; db/db mice simulate type 2 diabetes in human. Vasculopathy in different organs appears over a period of time, In C57BL/6 mice Streptozotocin induced in C57BL/6 mice develop typical microangiopathy mostly at 8‒10 weeks after the onset of DM and develop advanced microangiopathy at 12–16 weeks. db/db mice develop microangiopathy at about 20 weeks of age and develop advanced microangiopathy at 34 weeks

The natural course of DPDs in rodents from the same genetic background would provide insights into the pathological changes in various tissues at the same time. Streptozotocin induced in C57BL/6 mice develop microangiopathy in various organs at 4‒20 (mostly 8‒10) weeks after the onset of DM;^308–310^ db/db mice simulating type 2 diabetes develop microangiopathy in various organs at 16‒40 (mostly 20) weeks of age.^311,312^ The temporal progression of diabetic microvascular complications in mice are Concurrent regardless of the modeling modality (Fig. 6). DKD is the earliest to appear, with peripheral motor nerve conduction velocity decelerating at 4 weeks of DM (C57BL/6 mice).^313^ Microvascular function in diabetic mice partially changes around 8–10 weeks after DM onset (C57BL/6 mice)^310,314–318^ or 20 weeks of age (db/db mice),^268,311,319,320^ manifested as pathological staining of various organs changes. After 8 weeks of hyperglycemic state, further pathological changes of diabetic C57B/6 mice in microvascular structure can occur, including expansion of the mesangial matrix of renal microvasculature and rupture of the renal tubular epithelium^321^ The retinal ultrastructure changes, infoldings in retinal pigment epithelium layers are decreased, and balance or proprioception are impaired due to neurovascular disease.^322^ At 12‒20 weeks of DM, late panvascular disease can occur, with advanced vasculopathy of the heart, retina, or brain.^323–325^ occurring slightly before that of the kidneys.^326^ Diabetic macroangiopathy does not develop spontaneously in rats after DM modeling; rather, it is generally induced through specific diet control or vascular occlusion of the DM model rats.^327^ Thus, after simply constructing animal models of diabetes, studies on microangiopathy and macroangiopathy in the same models are scant. Rodents have different lipoprotein metabolism from humans and are highly resistant to AS. C57BL/6 mice are relatively too resistant to intraperitoneal injections of STZ to construct a model of DKD, and various diabetic complications require different animals for optimal modeling. Therefore, the optimal animal model for DPD studies awaits further investigation and clinical evidence.

**Fig. 7:**
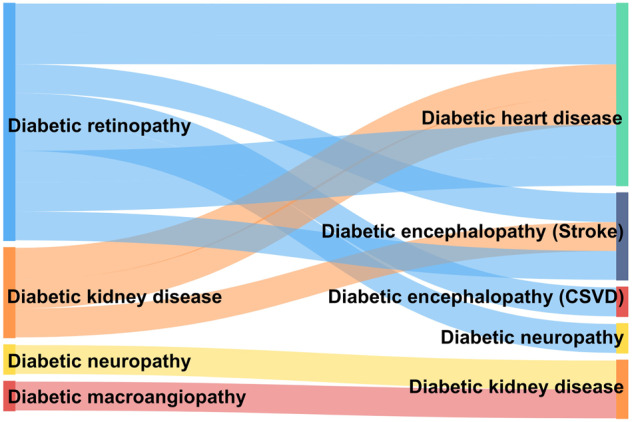
Predictive relationships among DPDs. CSVD cerebral small vessel diseases

Relationships among DPDs (Fig. 7) outcomes in vital organs (heart, kidney, and brain) are mutually predictive. DR is associated with coronary atherosclerotic heart disease, macrovascular events (including stroke), and all-cause mortality.^328^ Screening retinal microvessels have a potential role in the risk identification of cerebrovascular and neurodegenerative diseases.^329^ In T2DM, retinal parameters and a genome-wide polygenic risk score for coronary heart disease have independent and incremental prognostic value compared with conventional cardiovascular risk assessment.^330^ Risk of macrovascular complications in patients with diabetes and retinal artery occlusion for at least 5 years after an obstruction event is increased compared with those who do not have such occlusion. Therefore, retinopathy can predict cardiovascular risk in patients with T2DM.^331^ DR is closely associated with stroke^332–336^ and cerebral microangiopathy.^337^ During embryonic development, the diencephalon is homologous to the retina and optic nerve, especially the capillary-linked microglia and neuronal synapses, which are abundant in the retina and brain and sensitive to blood glucose.^338^ The microvasculature of the retina and the axonal function of retinal ganglion cells can be detected using fundus photography and optical coherence tomography to assist with the diagnosis of cerebrovascular neuropathy.^339^ Retinal microvascular imaging findings closely correlate with cerebral infarction and white matter lesions, parapapillary retinal nerve fiber layer thickness, macular thickness, and volume being indicators of stroke risk.^340^ Changes in the retinal vasculature can predict various stroke subtypes, suggesting that retinal vascular changes reflect specific cerebral microangiopathy and might even distinguish stroke from other causes of focal neurological deficits.^341^ In contrast, qualitative retinal vascular signs and quantitative retinal vascular measurements of narrowing small retinal arteries and widening small retinal veins, might indicate a cognitive decline.^342^ Compared to that with stroke, the association of DR with dementia and cognitive decline is more limited, suggesting a need for further prospective studies. In addition, because risk factors for stroke differ between patients with and without diabetes, stroke risk prediction models should include data on DR and DKD,^335^ which is a topic for future studies. DKD increases the risk of stroke, cerebral infarction, and cerebral hemorrhage.^334^ However, some subclinical cardiovascular complications of DM are not associated with stroke.^343^ Retrospective studies have shown that DR is associated with the development of DKD and the decline of renal function.^344–346^ Subsequent retrospective studies have found a positive association between DR and the risk of DKD progression.^347,348^ Retrospective findings of narrowing small retinal arteries and widening small veins both suggested the development of DKD,^349^ and this was later confirmed by a cross-sectional study^350^ and several prospective studies.^351–353^ Diabetic macroangiopathy and neuropathy are also important risk factors for DKD. Prospective studies have shown that carotid plaques and aortic stiffness are associated with DKD progression.^354,355^ A retrospective cohort study found that coronary artery calcification played a similar predictive role.^356^ Two retrospective cohort studies found that peripheral neuropathy and cardiac autonomic neuropathy are strong predictors of DKD.^357,358^ As discussed, vasculopathy due to various diseases, especially DR, is generally predictive of DKD progression, providing evidence supporting the panvascular nature of DM.

### Molecular mechanism and signaling pathway of DPDs

#### Cellular energy metabolism

Cellular energy metabolism requires energy supply from glucose, fatty acids, amino acids, etc.^359,360^ In the diabetic state, abnormalities in cellular energy metabolism affect macrovascular and microvascular lesions,^361–363^ including abnormalities in substrate delivery to vascular EC or target organ cells (e.g., cardiac myocytes, thylakoid cells in glomeruli, and neurons and Schwann cells in peripheral nerves), conversion of the ratio of cell-specific fuel sources between glucose intermediates, fatty acids, and amino acids, changes in respiratory chain protein function, and uncoupling of respiratory chains.^364–366^ The hyperosmolar state and abnormal energy metabolism of diabetes increase the PKC pathway, endothelial xanthine oxidase, and the eNOS uncoupling pathway, promoting increased ROS levels.^367,368^ The mechanisms of energy metabolism imbalance vary slightly between target organs with different mitochondrial content and different major energy supply substances.^369^

##### Glucose metabolism

**Fig. 8:**
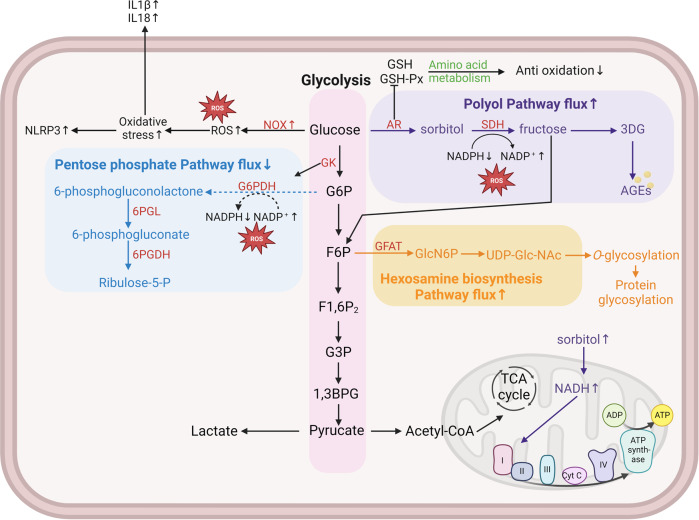
Schematic diagram of cellar sugar metabolic pathways. Cells obtain energy through multiple gluconeogenic pathways. These include glycolysis, polyol, hexosamine, and pentose phosphate pathways. In the diabetic environment, excessive intracellular glucose causes abnormal activation of the polyol and hexosamine pathways, and inhibition of the major glycolytic and pentose phosphate pathways, resulting in continued accumulation of reactive oxygen species, which ultimately leads to increased oxidative stress loss in cells and induces the development of DPDs. Different metabolic pathways are distinguished by different colors, with pink representing the glycolytic pathway, purple representing the polyol pathway, blue representing the pentose phosphate pathway, and yellow representing the hexosamine pathway. All enzymes are indicated in red. Solid arrows indicate that this process is promoted and dashed arrows indicate that this process is inhibited. IL interleukin, GSH glutathione, GSH-px glutathione peroxidase, AR aldose reductase, SDH sorbitol dehydrogenase, 3DG 3-deoxyglucosone, AGE advanced glycosylation end, NOX reduced nicotinamide adenine dinucleotide phosphate oxidase, ROS reactive oxygen species, NLRP3 NOD-like receptor thermal protein domain associated protein 3, GK glucokinase, G6P glucose-6-phosphate, F6P d-fructose-6-phosphate disodium salt hydrate, F1,6P2 fructose 1,6-bisphosphate, G3P glyceraldehyde 3-phosphate, 1,3BPG 1,3-bisphosphoglycerate, G6PDH glucose-6-phosphate dehydrogenase, 6PGL 6-phosphogluconolactonase, 6PGDH 6-phosphogluconate dehydrogenase, GFAT glutamine-fructose-6-phosphate aminotransferase, TCA tricarboxylic acid cycle

Glucose metabolism is the main factor affecting cellular energy metabolism. The “unification hypothesis”^226^ suggests that several seemingly independent biochemical pathways that are overactivated in diabetes are actually caused by excessive intracellular glucose flux (Fig. 8). EC are extensively damaged in DPDs, and EC produce ATP mainly by glycolysis.^370^ In hyperglycemic states, changes in the metabolic pathways of sugar (increased flux of the hexosamine pathway, increased flux of the polyol pathway, decreased flux of pentose phosphate and glycolytic pathways) lead to decreased production of NADPH and increased ROS, exacerbating oxidative stress.^371^ In the pentose phosphate pathway, glucokinase/hexokinase is involved, and this enzyme also regulates glucose transport into cells, as well as glycogen metabolism and gluconeogenesis. Activation of glucokinases (Dorzagliatin, PB-201, AZD-1656, etc.) has a regulatory effect on glucose homeostasis,^372–377^ but no additional vascular protective value has been reported yet.^378,379^

Hyperglycemia reduces glucose-6-phosphate dehydrogenase (G6PDH)-mediated entry of glucose into the pentose phosphate pathway, but rather into the polyol pathway through conversion to sorbitol by the rate-limiting enzyme AR, and these processes are accompanied by a decrease in the rate of NADPH (an important intracellular reducing agent) production.^380^ Meanwhile, high glucose induces activation of NADPH oxidase (NOX) to produce ROS, increases oxidative stress levels, and promotes NLRP3/IL-1β and IL-18 to increase inflammation levels.^381^ Diabetes promotes the accumulation of fructose-6-phosphate (F6P), which can lead to an increase in the hexosamine (HBP) pathway and overproduction of UDPn-acetylglucosamine.^382^ UDPn-acetylglucosamine is involved in intracellular protein regulation, especially for post-translational modifications of proteins such as O-GlcNAcylation.^383^ The HBP pathway accounts for a small percentage of glucose metabolism and does not affect tissue energy supply, but regulates glucose transporter protein and insulin signal transduction, regulates glycogen synthesis and elevates cellular O-glycosylation levels, stimulates cytokines, etc.

The sorbitol/polyol pathway is similarly increased in the high glucose state, where sorbitol is, in turn, converted to fructose by sorbitol dehydrogenase (SDH), resulting in the production of 3-deoxyglucosone (3DG), a highly reactive α-oxo-aldehyde, and promoting the production of AGEs.^384^ AR is also a key enzyme in this pathway. AR promotes the conversion of glucose to sorbitol and further to fructose with the participation of NADPH (produced by the pentose phosphate pathway). In the hyperglycemic state, AR depletes NADPH while increasing the accumulation of F6P, so there is a close influence between several glucose metabolic pathways. Not only that, AR decreases glutathione levels and glutathione peroxidase activity, and reduces cellular antioxidant capacity through amino acid metabolism. Elevated intracellular sorbitol also provides excess nicotinamide adenine dinucleotide (NADH) to the mitochondrial electron transport chain, which is a substrate for complex I (substrate for complex I) in mitochondria and closely affects mitochondrial function and cellular energy metabolism.^385^ Overexpression of AR in mice increases susceptibility to diabetes-induced AS and ischemia/reperfusion injury.^386,387^ In contrast, aldose reductase inhibitors (ARIs) and AR gene-deficient animals have vascular protective effects in DPDs.^83,388^ Some natural compounds and plant extracts have shown the inhibitory effect of aldose reductase 2 (ALR2), which can improve inflammation and protect the vascular endothelium, but in recent years ARIs have not been successfully available for widespread clinical use.^389^

##### Amino acid metabolism

Amino acid metabolism plays an important role in diabetes and complications, and branched-chain amino acids may serve as new biomarkers as well as signaling pathways suggesting the risk of DPDs.^390,391^ Glutamine is a branched-chain amino acid that is synthesized by glutamine synthetase (GS) and hydrolyzed by glutaminase (GLS).^392,393^ Glutamine levels were negatively associated with BMI and insulin resistance index (HOMA-IR) in men with type 2 diabetes.^394^ Glutamine in plasma binds the bridging protein GRB-10^394^ and improves the insulin resistance of cells. It also promotes the secretion of GLP-1 and GLP-2 secretion from intestinal cells.^395,396^ Vascular EC expresses glutaminase (GLS) and break down glutamine, and glutamine deficiency or inhibition of endothelial GLS1 can cause impaired EC proliferation and reduced vascular neogenesis.^397^ Increased glutamine synthetase (GS) transcription or increased glutamine levels promote macrophage polarization and atherosclerotic plaque formation.^398^ Chromosome 1q25 single nucleotide polymorphism (SNP) variants cause reduced GS expression in EC. In these populations, patients with diabetes have an increased risk of CAD but not patients with diabetes, and the causal mechanism remains to be determined.^399^

Glutamine metabolism in cells holds two branches: glutamine catabolism and asparagine synthetase/gamma-aminobutyric acid (ASNS-GABA) shunting. These two pathways independently regulate the AMP-activated protein kinase/mechanistic target of the rapamycin kinase complex (AMPK/mTORC) pathway, mediating cellular autophagy.^400,401^ Also, glutamine provides anaplerotic substrates for the TCA cycle.^397,402^ In EC, 30% of the tricarboxylic acid carbon comes from glutamine, comparable to that produced by the glycolytic pathway, and the novel hypoglycemic agent SGLT-2i can regulate mitochondrial oxidative phosphorylation and improve cellular energy metabolism through the above pathway.^403^ Glutamine metabolites are involved in intracellular oxygen reduction regulation. EC produces glutathione via glutamine, which regulates redox homeostasis, and its depletion makes EC vulnerable to ROS-induced damage. Glutamine catabolism produces glutamate that is converted to ornithine^404^ and aspartate^397^ and these generated amino acids are also involved in the proliferation of EC.^405^ EC proliferation is reduced after silencing of asparagine synthetase (ASNS), and supplementation with asparagine and α-ketoglutarate reverses the EC damage caused by glutamine deficiency.^397,401^ Thus, asparagine is also an important part of amino acid metabolism in DPDs.^406^

Amino acid metabolism also affects oxidative stress in EC via the arginine metabolic pathway.^407^ Vascular protective NO is produced via endothelial-type nitric oxide synthase (eNOS).^408,409^ In vitro experiments, arginine deficiency leads to EC eNOS dysfunction.^410^ Elevated arginase levels lead to L-arginine depletion, decreased output of NO, increased ROS, and impaired endothelium-dependent vasodilation.^411^ On the other hand, EC can absorb serine directly or produce serine in the reaction intermediate 3-phosphoglycerate of the glycolytic pathway^412^ for nucleotide biosynthesis and redox homeostasis.^413^ The serine pathway is activated in the high glucose state and it synergizes with the pentose phosphate pathway with single carbon metabolism to alter chromatin status and promote inflammation.^414^

##### Fatty acid metabolism

Diabetic patients often have abnormal lipid metabolism, and hyperlipidemia can lead to increased cellular uptake of fatty acids through passive diffusion and protein-mediated pathways. A cluster of differentiation 36 (CD36) and the fatty acid binding protein (FABP) family mediate fatty acid uptake into tissues,^415^ and soluble CD36 expression is increased in diabetic patients.^416^ Fatty acid uptake and transport by EC is extremely important for many cellular processes, including membrane synthesis, intracellular signaling, ATP production, and post-translational modification of proteins.^417^ Imbalance of fatty acid metabolism in EC does not lead to significant abnormalities in energy supply or disturbance of redox homeostasis, but can impair de novo nucleotide synthesis for DNA replication.^418^ For example, DNA repair factors such as Polyadp-ribose polymerase (PARP) are involved in fatty acid metabolism.^419^ In a high glucose state, vascular damage can be aggravated by PARP1.^420^

Fatty acid metabolism plays an important role in high-energy-demand cells and is closely associated with diabetic heart disease in DPDs.^421,422^ Overexpression of GLUT-1 in the myocardium increased glucose levels in cardiomyocytes and revealed that FAO in the heart was inhibited and that a high fatty acid diet failed to upregulate FAO in these hearts, while glucose supply was significantly increased, further leading to activation of p38 mitogen-activated protein kinase and increased oxidative stress in the heart.^423^

#### Mitochondrial dysfunction

An imbalance in energy metabolic pathways causes impaired mitochondrial function, most often manifested as increased mitochondrial autophagy and ROS production.^424^ Mitochondrial function plays an important role in cellular energy homeostasis. Alterations in glycolytic pathways, fatty acid oxidation, and some amino acid metabolism in the high glucose state can affect mitochondrial oxidative phosphorylation processes. AGEs production in the hyperglycemic state and AGE-RAGE-induced increase in cytoplasmic ROS promote the production of mitochondrial superoxide and the development of diabetic microangiopathy in the condition of hyperglycemia.^425,426^ Most ROS are derived from complexes I and III in mitochondria.^427^ In addition to complexes I and III, the NOX family also promotes the mitochondrial production of ROS.^428,429^ NOX4 is the highest expressed member of the NOX family and is upregulated by a variety of agonizts and cellular stress.^429,430^ Administration of novel mitochondria-targeted drugs helps to improve the mitochondrial ROS/NLRP3 axis and attenuate mesangial tubular injury in DKD;^431^ similar manifestations exist in the myocardium, attenuating diabetic myocardial ischemia-reperfusion injury68. The intramitochondrial protein p66Shc can promote increased reactive oxygen species in mitochondria by interfering with Ras activation or binding to cytochrome C and other.^432^

Mitochondria can store calcium ions and act in concert with the endoplasmic reticulum and extracellular matrix to control the dynamic balance of calcium ion concentration in cells and regulate the cell cycle and apoptosis.^433^ High glucose can affect myocardial contractile function by upregulating sarcolipin (SLN) promotes calcium sparks.^434^ Therapeutically, metformin inhibits mitochondrial respiratory chain complex-1 and regulates cellular energy metabolism.^435^ In addition, metformin and GLP-1 agonizts regulate the glucagon-lowering hormone glucagon-like peptide-1 and the bile acid pathway and alter the composition of the gut microbiota, which may also indirectly affect mitochondrial function.^362,436^

Mitochondrial energy metabolism differs among target organs, and myocardial mitochondrial content is abundant. Diabetic mice have altered mitochondrial function in the myocardium earlier than in the kidney, brain, and liver.^437^ Further in-depth mechanistic exploration is worthwhile in tissues with high mitochondrial content.

#### Insulin resistance

Insulin resistance is the most common and widespread molecular mechanism of diabetic complications, not only in the high glucose state with extensive activation of insulin receptor signaling pathways, such as the regulation of glucose uptake by GLUT.^438^ Even in the presence of normal blood glucose, insulin resistance is still harmful, and the lack of insulin receptor signaling pathways in renal pedal cells induces a disease state similar to diabetic nephropathy.^439^

The most common downstream mechanisms of insulin resistance include inhibitory phosphorylation of IRS, growth factor receptor binding protein-2 (GRB-2), GRB-10, SHC transforming protein (SHC), and SH2B adapter protein-2 (SH2B-2) through induced insulin receptor.^439,440^ Selective glucose transporters exist in different target organs, such as the renal SGLT2 receptor;^441^ the distribution of GLUT receptors in different target organs and the pathways also differ.^78,442^ This paper summarizes the new advances in insulin signaling receptor pathways and their roles from 2018 to date (Table 4).

**Table 4:** List of novel targets with emerging implications related to insulin receptor signaling

Insulin receptor signaling	Target tissue	Effect/ potential role	Reference
HCF-1	Hepatocyte (human, mice)	HCF-1-dependent pathway regulating Glucose and lipid metabolism	^516^
IRS/PTP1B	Cerebral microvascular endothelial cells (mice)	Hyperinsulinemia affects insulin receptor signaling and internalization of endothelial cells	^517^
SMPDL3b/C1P/Cav-1	kidney cortexes (rats), podocytes (human)	Inducing glucose and lipid metabolism, protein synthesis	^518^
IRS/IGF1/SRF	Myocardium (mice)	Affecting autophagy and apoptosis of cardiomyocytes	^519^
SDF-1/CXCR4	Primary endothelial cells (human), CD31 + cells (mice)	Affecting immune cell recruitment to the vascular wall or tissue parenchyma	^520^
miR-15b, miR-16, miR-30b/IRS2	Endothelial cells (human)	IRS2 and eNOS in ECs are ceRNAs and related to the Akt signal pathway	^521^
IRS	Renal hemodynamics (mice)	stimulation of renal functions and renal hemodynamics	^522^
IRS/p53/KLF4	Vascular smooth muscle cells (mice)	IRS-1/p53 affects the progression of atherosclerotic lesions in hyperglycemia	^523^
QKI-7	Endothelial cells (human)	Promotes mRNA degradation of essential genes for EC function	^524^
EPDR1	Endothelial cells (mice)	Mediate pathological angiogenesis during hyperinsulinemia and insulin resistance	^525^
IRS-1 rs956115 genotypes	(Human)	IRS-1 CG/GG genotype are at higher risk of major adverse cardiovascular events	^526^
**Therapeutic effect**			
Amlexanox inhibition of TBK1/IKKe	Serum (human)	Lowering HBA1c, fructosamine, ameliorates metabolic dysfunctions	^527^
Loganin inhibition of JNK-IRS-1/Akt/GSK3β	Peripheral nerve (rats), SH-SY5Y cell (human)	Inhibiting oxidative stress-provoked inflammation, improved Nerve pain behaviors	^528^
SGLT-2i inhibition of JunD/PPAR-γ, IRS-1, IRS2	Endocardiomyocytes (human)	Inhibiting JunD/PPAR-γ overexpression and lipid accumulation, ameliorate diabetic cardiomyopathy	^529^

*C1P* ceramide-1-phosphate, *IRS* insulin receptor substrate, *PTP1B* protein tyrosine phosphatase, non-receptor type 1, *SMPDL3b* sphingomyelin phosphodiesterase acid-like 3b, *Cav-1* caveolin-1, *IGF1* insulin-like growth factor 1, *SRF* serum response factor, *SDF-1* stromal cell-derived factor-1, *CXCR4* CXC receptor 4, *CD31* endothelial cell adhesion molecule-1, *eNOS* endothelial nitric oxide synthase, *ECs* endothelial cells, *ceRNAs* competing endogenous RNAs, *Akt* protein kinase B, *KLF4* Krüppel-like factor 4, *QKI-7* quaking-7, *EPDR1* ependymin-related protein-1, *TBK1* TANK binding kinase 1, *IKKe* IkappaB kinase, *HBA1c* glycated hemoglobin, *JNK* c-Jun N-terminal kinase, *GSK3β* glycogen synthase kinase-3β, *JunD* jund proto-oncogene subunit

#### Glycosylation end-products

As the end-products of dysfunctional EC metabolism, AGEs have profound effects on the immediate extracellular environment of EC as well as on other cell types. AGEs can bind key proteins (such as laminin, elastin, and collagen) in the extracellular matrix (ECM) basement membrane, leading to increased vascular stiffness promoting DPDs.^443–445^ AGEs may also affect coagulation and hemodynamics, cause increased vascular permeability and induce tissue factor expression.^446,447^ The extensive intracellular action of circulating AGEs is mediated through the attachment of receptors for RAGE, which is expressed in monocytes, smooth muscle cells (SMC), and EC.^448,449^

RAGE induces an inflammatory cascade response by activating the transcription factor NF-κB, which promotes the expression of growth factors and adhesion molecules. On the other hand, RAGE activates NADPH oxidase to increase oxidative stress; RAGE also binds to tissue-specific proteins to promote local vascular injury,^450,451^ such as binding AGEs or S100/calmodulin or β-amyloid peptide to exacerbate cerebral hemorrhage;^452^ RAGE binds to EC to promote increased NADPH expression leading to inflammatory responses and a prothrombotic state by activating diaphanous-related 1 (DIAPH 1), ERK1, ERK2, and PKC.^451,453^ AGE/RAGE on mononuclear macrophages increases CD36 expression, promotes OX-LDL uptake while decreasing high-density lipoprotein (HDL) efflux, and promotes foam cell formation; AGE/RAGE action on vascular smooth muscle cells (VSMC) induces autophagy through the ERK/Akt pathway^454^ and increases ROS and NOS levels through activation of NOX and NF-κB,^455,456^ increases oxidative stress, and accelerates atherosclerotic plaque progression.^447^ Excessive AGE formation and overactivation of the hexosamine pathway induce angiopoietin-2 transcription by inhibiting transcriptional co-repressor complex binding and silencing the angiopoietin-2 promoter.^457^ The formation of AGEs may partially explain the “hyperglycemic memory” of tissue damage, i.e., the vascular damage that occurs during hyperglycemia can continue into the normoglycemic cycle.^458^ Hyperglycemia may alter cellular epigenetics, such as DNA and protein modifications, DNA methylation, non-coding RNA, or histone modifications by receptor-mediated mechanisms to alter cellular function.^459^ Thus, epigenetics, as well as AGEs, are important targets for intervention outside of glycemic control.

### Prospects

DPD has become a major public health problem that requires urgent attention, as 11% of patients with T2DM complicated with AS have a multivessel disease.^460^ Patients with T2DM complicated with the multivessel disease have a significantly higher risk of ischemic events and overall mortality than those with T2DM with single-vessel disease. The significant cardio-renal benefits of metformin and SGLT-2i beyond glycemic control have led to the emergence of an integrated treatment model that can be applied to manage DPD.^461^ The pathogenesis of DPD involves insulin resistance, inflammation, oxidative stress, and AGEs, with a wide prevalence of hyperglycemic “metabolic memory” and some epigenetic alterations in various vascular pathologies.^462,463^ The latest drugs in development are ARIs, tyrosine phosphatase 1B inhibitors, PPAR-γ agonizts, regulators of the glucagon system, and mitochondrial energy modulators. Antisense oligonucleotides and monoclonal antibodies are widely developed.^464,465^ New biomarkers and drug intervention targets to improve the prognosis of DPD hold enormous potential for clinical applications.

Diabetic microangiopathy and macroangiopathy significantly differ, and simultaneously developing them in animal models is challenging. However, clinical practice has often found coexisting interactive macroangiopathy and microangiopathy.^303^ The predictive relationship among DPDs also suggests that diabetic microangiopathy is not the only indicator of the risk for macroangiopathy, and that the various types of diabetic microangiopathy are also closely interrelated.^460^ In recent years, several molecular pathways with simultaneously large/microvascular protective effects have been identified (PPAR-γ, CXCR4, etc.), but most of them are still in animal experiments or phase I clinical stage.

DPD research also faces several challenges. Concepts should be integrated across disciplines that focus on specific aspects of DM and angiopathy management in different organs. Research results of new technology might be redundant. For example, miRNA-based studies of diagnosis or regulation have found that miR-21 plays an important role in diabetic vascular complications;^211,273,466–470^ however, the role of miR-21 in organs and at various points during disease progression remains unknown because concepts across disciplines have not been fully integrated. New technologies such as induced pluripotent stem cells should be developed to enrich experimental findings of DPD and construct model human blood vessel organoids.^129^ Multi-omic studies^471,472^ should explore the pathogenesis of DPD. Furthermore, new cross-disciplinary, integrated prevention and treatment models should also be developed. Cross-collaborations among medical and research institutions and academic organizations are needed to narrow gaps among research results, new technologies, and clinical applications. Cross-collaborations among medical institutions at all levels are needed to fully integrate the resources of primary- and higher-level care. Cross-collaborations among doctors, nursing staff, and patients are needed to ensure personalized treatment for patients. Cross-collaborations among clinical disciplines, including endocrinology, cardiovasology, geriatrics, neurology, nephrology, vascular surgery, and nutrition are also needed to integrate comprehensive evidence. Traditional Chinese medicine also has considerable potential for the prevention and treatment of DPD.^473,474^ A comprehensive DPD prevention and treatment system should be established with Chinese characteristics, promoted cooperation, and accelerated clinical translation to improve the overall prevention and control of chronic diseases such as diabetes.
